# A Preventive Approach to Arsenic Toxicity: Testing Folic Acid in Bangladesh

**DOI:** 10.1289/ehp.123-A306

**Published:** 2015-12-01

**Authors:** Carol Potera

**Affiliations:** Carol Potera, based in Montana, also writes for *Microbe*, *Genetic Engineering News*, and the *American Journal of Nursing*.

Chronic arsenic exposure affects 35 million people in Bangladesh, where arsenic in drinking water runs up to 100 times the World Health Organization’s limit of 10 µg/L.^1^ Among folate-deficient Bangladeshi residents who drank well water contaminated with arsenic, researchers previously reported that folic acid (FA) supplementation at the recommended daily allowance of 400 µg for 12 weeks was associated with an average 14% reduction in blood arsenic levels.^2^ In this issue of *EHP*, members of that team report further evidence that FA supplementation may be an effective intervention for arsenic-exposed populations.^3^

The study was conducted as part of the Health Effects of Arsenic Longitudinal Study, a prospective cohort study in Araihazar, Bangladesh. In a 24-week randomized trial, 622 participants received one of five treatments: 400 µg FA, 800 µg FA, 3 g creatine, 3 g creatine with 400 µg FA, or placebo. Halfway through the trial, some of the participants who received FA treatments were switched to placebo to test whether blood arsenic would revert to pre-intervention levels as arsenic was released from tissue stores. All the participants, who came from households where the water contained at least 50 µg/L arsenic, received water filters at enrollment.^3^

**Figure:**
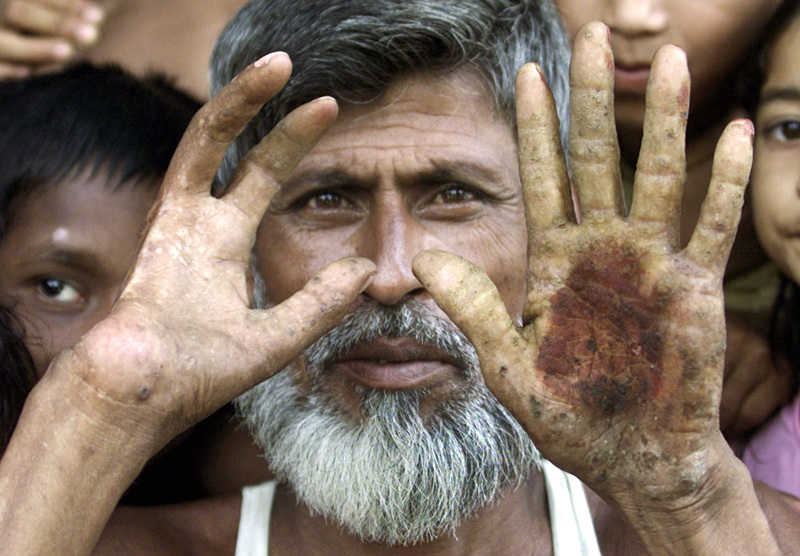
Skin lesions are a common result of chronic high exposures to arsenic in drinking water. In addition to the severe lesion on his left hand, this Bangladeshi man lost two fingers on his right hand to arsenic damage. © Rafiquar Rahman/Reuters

Folate facilitates the addition of methyl groups to inorganic arsenic, and these methylated forms can be excreted in urine.^4^ Methyl groups are also used within the body to produce creatine, a source of energy for muscle cells. If people get creatine from food or dietary supplements, methyl groups that would have been used to produce endogenous creatine are freed up for other chemical reactions, such as arsenic methylation^5^—hence, the creatine supplementation in this study.

At 24 weeks, people taking 800 µg FA, including those who switched to placebo halfway through, showed an average decline in blood arsenic levels of 12%, compared with 2% for those taking placebo for 24 weeks.^3^ Based on a previous study of blood arsenic and incidence of arsenic-related skin lesions,^6^ the researchers estimated that a 12% lower average blood arsenic level would result in a roughly 8.2% lower incidence of skin lesions.^3^

The people taking 400 µg FA alone or combined with creatine had blood arsenic levels similar to those of the placebo group,^3^ perhaps because few of the study participants were folate deficient, compared with all the participants in the earlier work.^2^ The researchers also are still analyzing arsenic metabolites, says study leader Mary Gamble, an associate professor of environmental health sciences at Columbia University’s Mailman School of Public Health. “It’s possible that arsenic methylation patterns changed in these groups,” she says, resulting in more easily excreted forms of arsenic.

The researchers found that the higher dose of FA lowered blood arsenic even in people who already had adequate blood levels of folate.^3^ This suggests that widespread fortification of food with folic acid may be beneficial, Gamble says, but formally testing this would require a large randomized trial. Dozens of countries currently fortify foods with FA; however, Bangladesh and many other arsenic-endemic countries do not.^7^

Chronic arsenic exposure increases the risk for heart disease and cancers of the skin, liver, prostate, lung, and bladder.^8^ Reducing exposure to arsenic via drinking water is needed to prevent arsenic-induced illnesses. But in addition to that goal, says Bernhard Hennig, a professor of nutrition and toxicology at the University of Kentucky who was not involved with the study, “nutritional interventions may offer a sensible way to prevent diseases associated with environmental insults.”
